# Voice Handicap Index evaluation in patients with moderate to profound bilateral sensorineural hearing loss

**DOI:** 10.1590/S1808-86942010000100011

**Published:** 2015-10-17

**Authors:** Felipe Barbosa Madeira, Shiro Tomita

**Affiliations:** 1 MD (Otolaryngologist); MSc student - Graduate Program in General Surgery/Otolaryngology - Federal University of Rio de Janeiro (UFRJ); 2 Full Professor - Medical School of the Federal University of Rio de Janeiro (UFRJ), Head of the Otolaryngology Department - Clementino Fraga Filho University Hospital Federal University of Rio de Janeiro (UFRJ)

**Keywords:** voice disorders, hearing loss, quality of life, questionnaires, voice

## Abstract

Voice and speech are regulated by hearing. Vocal disorders in patients with hearing loss have not been evaluated yet as to the subjective degree of disability they cause in this group.

**Aim:**

to compare the results of the Voice Handicap Index (VHI) obtained for patients with normal hearing and moderate to profound bilateral sensorineural hearing loss. Study design: Controlled, cross-sectional.

**Materials and Methods:**

A total of 76 adult patients being treated on a University Otolaryngology center were enrolled (38 with and 38 without hearing loss), ages ranging between 19 and 59 years, were asked to complete the Portuguese version of the VHI.

**Results:**

Total VHI score median values obtained were 23.5 and 4.0 for the study and control groups, respectively (p = 0.000). Significant differences between the two groups were found for all three VHI subscales (functional, physical and emotional) (p = 0.000).

**Conclusion:**

Our results lead us to infer a greater social and economical disadvantage as per assessed in the VHI of patients with moderate and higher bilateral sensorineural hearing loss.

## INTRODUCTION

Voice and speech production is a complex process involving numerous regulatory mechanisms.

The refinement and stabilization of such processes start in infancy; however they are only completed during adolescence, requiring motor information from the speech articulatory pathways (larynx, pharynx, oral and nasal cavities) as well as sensorial information. Such sensorial information is also obtained from a hearing feedback mechanism still not completely understood, used as information for the correction and/or improvement of muscle commands associated with vocal production, in such a way as to be able to achieve the necessary modifications and reach a certain vocal objective (for example, speak louder or slower).

In the year 2000, Perkell et al. published an extensive review on voice and speech motor control obtained from studies with hearing impaired individuals in comparison with normal-hearing individuals, starting a theory on the influence of hearing on these processes^1^. It is initially considered that each word is made up of sets of segments, represented as acoustic targets on the Central Nervous System (CNS). These targets are made up of multiple parameters, such as intensity (sound pressure level), fundamental frequency (F0), voice forming factors and the necessary articulatory patterns to reach such sounds. A continuous hearing feedback would characterize voice acoustics and speech pace, starting a mechanism which would revalidate speech production patterns already present in the CNS. Such parameters, called internal models, are acquired during infancy and adolescence, involving the relationship between vocal tract shape and movement, and their consequence on voice acoustic parameters. The most important functions of the auditory information obtained would be, besides maintaining the standards of this internal model, to control quickly and in a reflexive manner the parameters which affect speech intelligibility.

Voice characteristics such as the utterance of vowels and consonants, fundamental frequency and its variations are parameters studied in numerous occasions in order to investigate the role hearing plays on our voices. In a study published in 1990, Waldstein showed alterations in these voice parameters in individuals with postlingual profound hearing loss, when the internal voice and speech model would have already been acquired^2^. Through the speech evaluation of three adults with profound postlingual hearing loss in comparison to adults with normal hearing, Lane and Webster proved statistically significant alterations on F0 and on the pitch (perceptive hearing of the fundamental frequency and voice forming factors) of those individuals with hearing loss^3^.

In order to investigate the temporal characteristic of the voice change evolution caused by cochlear implants, five adult patients with profound postlingual hearing loss were analyzed at four different times: pre-implant; and at 1, 6 and 24 months after the implant^4^. Changes in vocal patterns were perceived right on the first assessment, following up to the second year after implant activation, when speech patterns from the patients fit normal patterns. Such results suggest that speech modifications after improvements on the hearing pattern would not be immediate, taking a progressive course which would depend on the hearing loss duration, the age of hearing loss onset and the quality of the hearing feedback.

Hamzavi et al. published a paper about the effects cochlear implants have on the vocal emission of patients with postlingual hearing loss with 10.1 years of hearing loss duration average time, and hearing onset mean age of 35.7 years, showing an improvement on the fundamental frequency pattern and on the consonant emission pattern^5^. In 1992, Perkell et al.^6^ also assessed patients with cochlear implants through the analysis of voice parameters and after implant activation and showed an increase on speech velocity and F0 improvement, and most of these alterations happened within 26 weeks. Comparable results were seen by Evans and Deliyski in 2007^7^, in three adults with prelingual loss, within a period of six months between the implant activation and the second assessment.

Svirsky et al. assessed two patients with cochlear implants through speech and with the implants on and off, considering the alterations which happened during this fast change in the auditory conditions of the tested indi-viduals^8^. The evaluations of both situations were carried out with intervals of minutes only, revealing voice sound pressure levels and blow voice levels. Alterations in the vocal pattern were more significant with the implant on than when it was off, in other words, the improvement observed after implant activation was more significant than the worsening which happened after it was turned off, indicating that the internal necessary model for normal speech production is maintained after removing the auditory feedback.

The characteristic of phoneme internal model maintenance is experimentally shown by Perkell through the analysis of the first and second voice forming factors emitted during vowel phonation by patients with postlingual hearing loss in situations of pre and post cochlear implant^6^. Vowel emission seemed not affected by the hearing loss, in other words, the internal phonation model would keep strong for a long time, even without hearing feedback.

The later the hearing loss establishes, the greater are the strength and permanence of the internal speech model^1^,^4^. During his analysis of seven patients with profound hearing loss, Waldstein showed that those with hearing loss of longer duration and those whose onset was at a younger age, showed greater deviations comparing the speech pattern of the controls in all the parameters assessed (vowels, consonants, F0 and its variation)^2^. Patients whose losses started at an adult age had alterations basically on the vowels' pattern. The internal model, having its development interrupted during childhood would not be mature enough to be promptly recalibrated by a hearing improvement.

The voice nasal pattern was also assessed as far as hearing is concerned. Nine patients with profound hearing loss submitted to cochlear implant were analyzed in five week intervals between implant activation and the second assessment, showing a significant improvement in F0 and its variation and that of nasality patterns^9^. Similar results in terms of nasality were published in 2008, in six children before and after the implant, and the author states that privation of hearing feedback could cause a difficulty in monitoring the velopharyngeal region valve mechanism^10^.

Here we showed the huge array of voice and speech alterations caused by profound sensorineural hearing loss. We can state that voice alterations are frequent in patients with hearing loss of such characteristics, especially because of changes in the hearing-dependant regulatory mechanisms. Given such fact, it is pertaining to assess how much these voice and speech alterations impact the lives of these individuals, which is not evaluated by these objective voice analyses.

Aiming at a better characterization and measuring of these difficulties experienced by the patients, many tools have been developed, mostly questionnaires involving voice-related quality of life. In a study published in 2005, the authors identified nine voice-related quality of life assessment tools^11^. The Voice Handicap Index (VHI) showed advantages regarding issues such as validity, practicality, versatility and information on the situation presented, besides being the objective, or the tool, to study the largest number of papers published among the questionnaires assessed.

The VHI is a questionnaire developed and published in 1997 by Jacobson et al. aiming at the self-assessment of the severity of the vocal alterations of patients with dys-phonia^12^. The term handicap means a social or economical disadvantage incurring from a disability of specific physical loss, in this case: vocal. It is made up of 30 questions, broken down into three groups according to the situation: functional, physical or emotional. Each one of these subitems has ten specific situations or questions, identified by their frequency of occurrence through a progressive numeric scale: 0 (never), 1 (almost never), 2 (sometimes), 3 (almost always) and 4 (always). We then obtain a partial scoring for each one of the three parameters and one total, the latter varying between 0 and 120. Such scorings are directly associated to the level of disability or restriction associated with the voice.

VHI was initially developed for the English Language. In 2004, Guimarães and Abberton published one of the first papers on VHI for Portuguese, by adapting to Portuguese the questions written in English^13^. Other papers with the VHI in Portuguese were also published: Jotz et al. led a study to check the accuracy of the VHI translated into Portuguese in order to differentiate dysphonic from non-dysphonic patients, showing that the VHI is a useful instrument to differentiate between the presence or absence of dysphonia as an assistant tool to diagnose and follow up the patients^14^.

VHI was used as a means to measure the voice-related handicap in different disease groups, such as organic dysphonias^15^, presbyphonia^16^, professional use of one's voice^17^,^18^, gastroesophageal reflux disorder (GERD) and laryngopharyngeal reflux disorders^19,^^20,^^21^, adduction and abduction spasmodic dysphonia^22,^^23,^^24^, thyroplasties^25,^^26,^^27^, microsurgery for benign^28^,^29^ and malignant^30,^^31,^^32^ disorders, radiography for laryngeal cancer^33^, use of tracheoesopha-geal^34^ and speech therapy^35^,^36^.

Numerous authors compared other methods of vocal assessment. In 2002, Hsiung et al. did the spectrographic analysis, and found poor correlations after the statistical tests. The same results were also obtained by other authors^29^,^37^: an objective method such as vocal spectrograph would not properly assess the subjective perception of the individual regarding his own voice. Other studies showed agreeing results concerning VHI improvement and voice acoustic patterns, as in patients submitted to laryngoplasty to treat unilateral vocal fold paralysis. The statistically significant improvement on voice acoustic patterns associated with VHI improvement showed that in some pathological cases there is agreement between the VHI and the vocal analysis objective patterns^25^, ^26^, [Bibr bib27][Bibr bib35][Bibr bib38].

Despite the extensive publication on voice and speech alterations associated with hearing loss of such characteristics, there is no report on the use of VHI to assess these complaints from patients with hearing loss. Also, there are no reports of hearing evaluation in patients with moderate hearing loss.

VHI analysis in patients with hearing loss could influence treatment: patients with higher VHI scores have to an early need to improve their audiometric thresholds, such as through the use of individual sound amplification devices (ISAD) or cochlear implants, depending on the intensity and cause of the hearing loss. Higher scores would also indicate the need for speech therapy, even in patients with moderate hearing loss. Such decisions must be made on a case-by-case basis, showing that VHI would also help in treatment customization and follow up of each individual, enabling a greater chance of treatment success and individual satisfaction.

The present study aims at assessing the different Voice Handicap Index (VHI) scores among patients with normal hearing and those with sensorineural hearing loss at a moderate level, checking the degree of loss the vocal alterations were causing to the latter group.

## MATERIALS AND METHODS

The present study was approved by the Ethics in Research Committee of the institution where it was done, under protocol # 234/06. This was a cross-sectional study, led between January of 2007 and July of 2008, with the individuals being recruited from the Otolaryngology outpatient ward.

Inclusion criteria were: age between 18 and 60 years; altered tonal and vocal audiometry, showing a bilateral sensorineural hearing loss of moderate degree at frequencies equal to and higher than 500 Hz for the group of individuals with hearing loss; normal tonal and vocal audiometry (audiometric thresholds below 25 dB on the frequencies tested) for the control group; videolaryngoscopy without alterations and obtaining a signed informed consent form from the patients.

Exclusion criteria involved: pre or perilingual hearing loss; alterations on voice auditory perception analysis; speech disorders (dyslalia, stuttering); professional use of the voice or its frequent use in an amateur way (to sing in churches and bands, for example); past of laryngeal surgery; hearing aid use at any moment prior to the assessment; past or current smoking habit and pulmonary or neurological disorders.

During our study period, we analyzed 96 patients with the audiometric profile matching those required in the study. After employing the exclusion criteria, thirty-eight of these patients were actually included in the study. Another group of thirty-eight individuals with normal tonal audiometries matched the study group people in age and gender were included as control group.

Videolaryngoscopy and the VHI were only carried out after the individual presented the researcher with the signed informed consent form. All the patients were submitted to vocal and tonal audiometry with the Intera-coustics CE10 audiometer, and the tritonal average was obtained for each ear separately through the mathematical average of the auditory thresholds in the frequencies of 500; 1.000 and 2.000 Hertz (Hz).

The hearing loss was classified in levels, for each ear separately and for both ears together (binaural) aiming at the descriptive analysis: moderate (between 41 and 60 dB), severe (between 61 and 80 dB) and profound (higher than 81 dB) hearing loss. Considering the two ears together, we used the following classification: moderate, moderate-to-severe, moderate-to-profound, severe, severe-to-profound and profound.

The laryngoscopy tests were done by the researcher with a 70 degree Storz rigid scope, Mini Xenon Storz light source, IK-CU43A Toshiba micro camera and 14 inch Sony monitor. In those cases in which the patients had an intense nauseous reflex preventing the test to be carried out, we used topical anesthesia with 10% lidocaine, if there were no contraindications related to it.

The patient him/herself must fill out the VHI questionnaire after being instructed about its content and how to fill it out, without the presence of the researcher or escorts. Originally translated into Portuguese by Guimarães and Abberton in 2004^13^, the questionnaire was adapted by the author of the present study in order to eliminate confounding factors for the patients with hearing loss (Chart 1). The sentence “I stopped using the telephone” was expanded to “I stopped using the telephone because of my voice” makes the true intention of the question clearer for the patient about his/her voice alteration. Only such item, number 4 of the functional subitem, needed to be adapted in such a way for this paper.

**Chart 1:** Voice Handicap Index (adapted from Jacobson et al.; 1997)

0 = NEVER 1 = ALMOST NEVER 2 = SOMETIMES 3 = ALMOST ALWAYS 4 = ALWAYS	

PART I: Functional aspect	

1) Do people have difficulties to understand your voice?	0 1 2 3 4
2) Do people have difficulties to understand you in noisy environments?	0 1 2 3 4
3) Does your family have difficulties hearing you when you call them at home?	0 1 2 3 4
4) Do you stop using the telephone because of your voice?	0 1 2 3 4
5) Do you avoid groups of people because of your voice?	0 1 2 3 4
6) Do you talk less to friends, neighbors and relatives because of your voice?	0 1 2 3 4
7) Do people ask you to repeat yourself when talking to you face-to-face?	0 1 2 3 4
8) Does your voice restrict you in your personal and social lives?	0 1 2 3 4
9) Do you feel left out in conversations or discussions because of your voice?	0 1 2 3 4
10) Has your voice problem caused you to lose your job?	0 1 2 3 4

PART II: Physical aspect	

1) Do you feel breathless when talking?	0 1 2 3 4
2) Does your voice vary during the day?	0 1 2 3 4
3) Do people ask: “What's wrong with your voice?”	0 1 2 3 4
4) Does your voice feel hissy or dry?	0 1 2 3 4
5) Do you struggle to produce your voice?	0 1 2 3 4
6) Is the clarity of your voice unpredictable?	0 1 2 3 4
7) Do you try to change your voice in order to sound different?	0 1 2 3 4
8) Do you make a lot of effort to speak?	0 1 2 3 4
9) Is your voice worse at the end of the day?	0 1 2 3 4
10) Does your voice fail in the middle of a conversation?	0 1 2 3 4

PART III: Emotional aspect	

1) Do you feel tense when talking to other people because of your voice?	0 1 2 3 4
2) Do people get irritated because of your voice?	0 1 2 3 4
3) Do you feel other people do not understand your voice problem?	0 1 2 3 4
4) Does your voice bother you?	0 1 2 3 4
5) Are you less sociable because of your voice?	0 1 2 3 4
6) Do feel impaired because of your voice problem?	0 1 2 3 4
7) Do you dislike it when people ask you to repeat yourself?	0 1 2 3 4
8) Do you feel embarrassed when people ask you to repeat yourself?	0 1 2 3 4
9) Does your voice make you feel incompetent?	0 1 2 3 4
10) Do you feel ashamed of your voice problem?	0 1 2 3 4

The statistical analyses were carried out using the SPSS 16.0 Statistical Package for Windows (SPSS Inc., Chicago, IL). Results were presented as median (minimum - maximum) and standard deviation (SD). The Mann-Whitney non-parametric test was utilized in order to compare the numeric variables between the groups (control versus patient). In order to study the correlation among the numeric variables we used the Spearman correlation's test (regarded as “r”). A p-value below 0.05 - considered statistically significant.

## RESULTS

The age median value for the group of patients was 50.5 years (SD = 10.9 years), with extreme values of 19 and 59 years. In the control group, the median was 48.0 years (SD = 9.9 years), with extreme values of 20 and 58 years. The analysis between the two groups in relation to age did not show any statistically significant difference (p = 0.344), confirming efficiency of the pairing by age range. There was a positive and statistically significant correlation in the control group between age and the left tritonal mean value (r = +0.35, p = 0.031), the physical VHI subitem (r = +0.42 p = 0.009) and total VHI (r = +0.35, p = 0.03).

**Graph 1:**
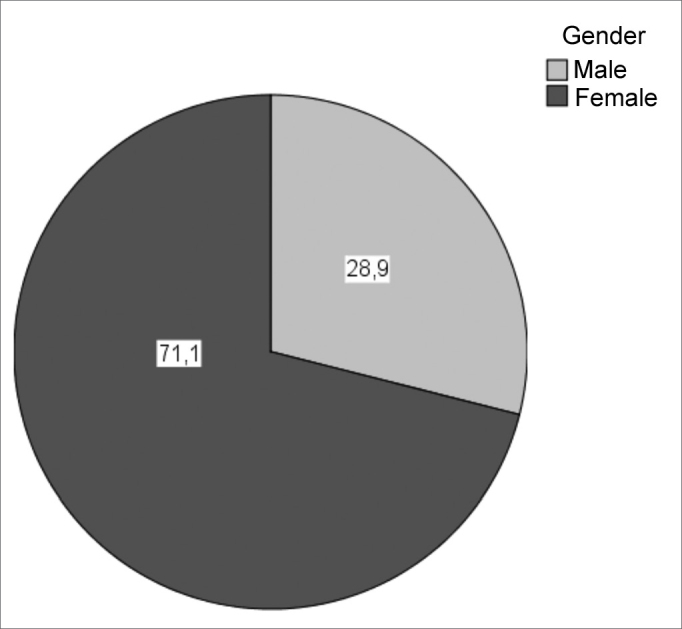
Distribution in percentage between the genders in the entire sample of individuals analyzed.

The distribution between patients and controls regarding gender was: 28.9% of males and 71.1% of females, keeping the same ratios because of pairing by gender in the samples (Graph 1). There was no correlation regarding gender with any of the items investigated in the two groups analyzed.

Following, we used the box-plot graph in order to show the data related to the right and left tritonal mean values and scores of the subitems and VHI total between the two groups studied. The horizontal line crossing the box translates the median for each group. The boxes have upper and lower thresholds corresponding to p25 and p75 of the sample, with the upper and lower lines connected to the box tracing the limits at p90 and p10, respectively. The circles show the outliers and the asterisks show the extreme values in these samples.

**Graph 2:**
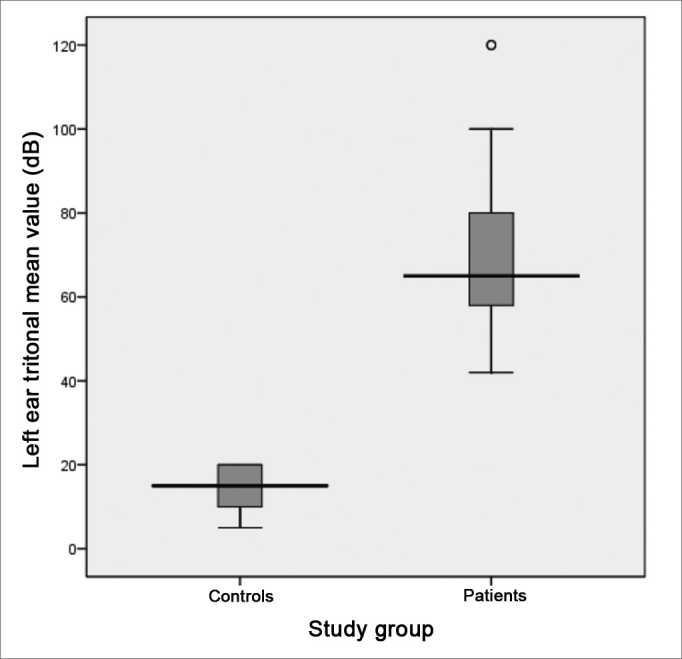
Distribution of the left ear tritonal mean values (dB) between the study groups (p = 0.000).

**Graph 3:**
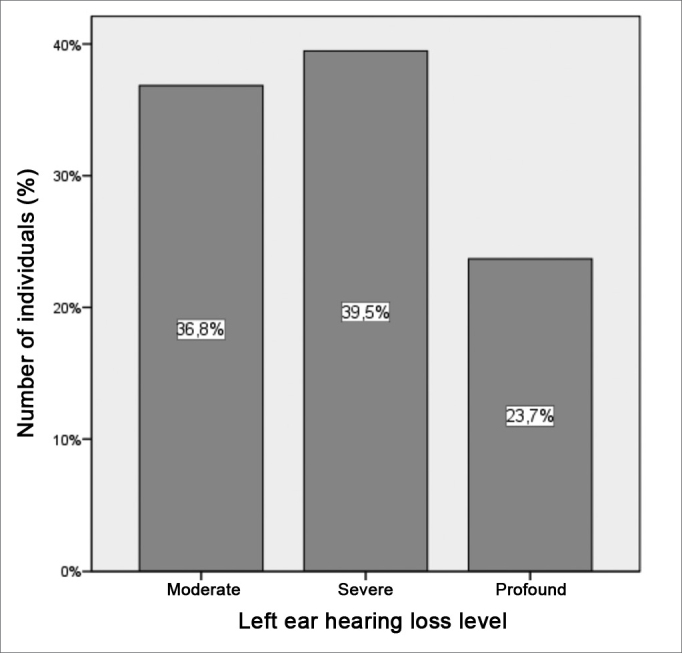
Distribution of the left ear hearing loss levels in patients with hearing loss.

The tritonal mean for the left ear showed medians of 65 (DP = 18.9 dB) and 15 dB (DP = 5.0 dB) between patients and controls, respectively with extremes of 42 and 120 dB for the patients and 5 to 20 dB among controls. The comparative analyzes between the two groups showed a statistically significant difference between the groups (p = 0.000) (Graph 2). It is descriptively shown the distribution of left ear hearing loss levels (Graph 3).

**Graph 4:**
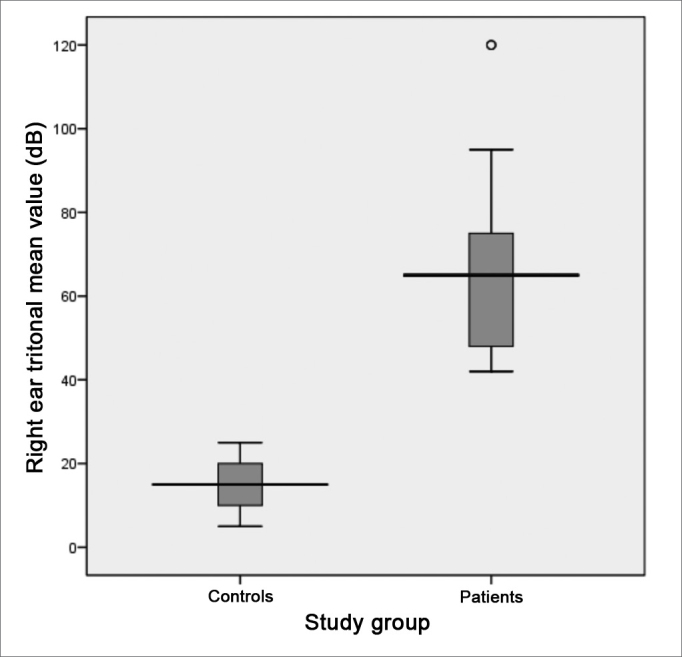
Distribution of the right ear tritonal mean values (dB) between the study groups (p = 0.000).

**Graph 5:**
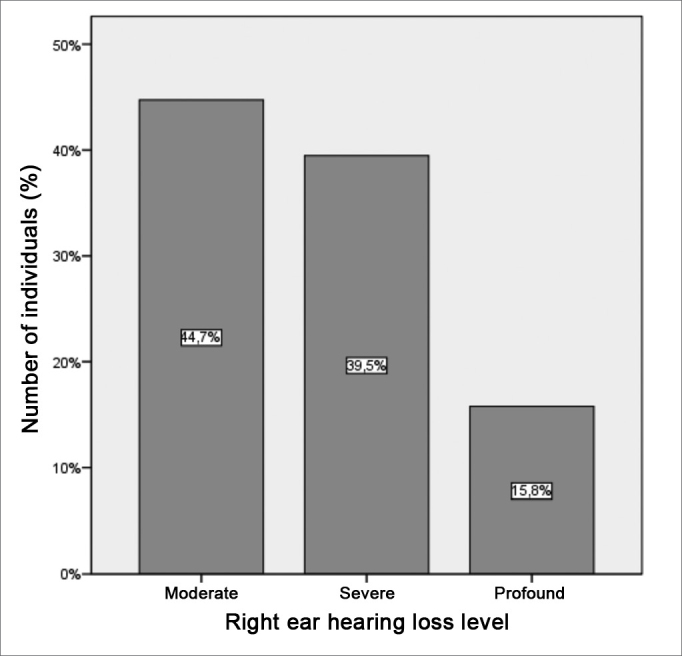
Distribution of the right ear hearing loss levels in the group of patients with hearing loss.

The tritonal mean value for the right ear also presented median values of 65 (SD = 19.9 dB) and 15 dB (SD = 5.1 dB) for patients and controls, respectively, with extremes of 42 and 120 dB for patients and 5 and 25 dB for controls. The comparative analysis between the groups showed a statistically significant difference between the two groups (p = 0.000) (Graph 4). It is descriptively shown the distribution of right ear hearing loss levels (Graph 5).

**Graph 6:**
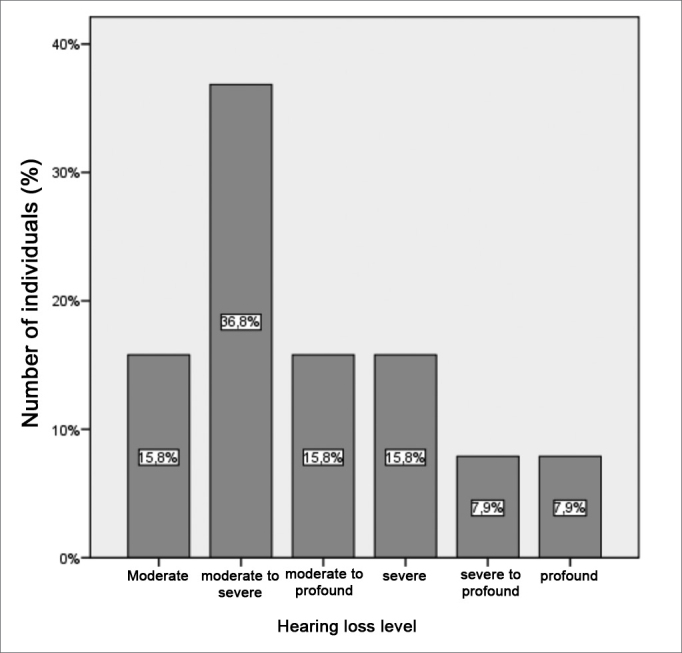
Distribution of the binaural hearing loss levels in the group of patients.

The analyses of data correlation with the levels of hearing loss only showed statistically significant difference when associated with the right and left tritonal mean values. The data associated with the binaural hearing loss levels are shown on Graph 6. We can see a predominance of patients with moderate to severe hearing loss (36.8%), followed by patients with moderate degree (15.8%) and moderate to profound (15.8%).

The hearing loss duration at the time of data collection showed a median of 14 years (SD = 11.1 years). The hearing loss duration extremes were from 1 to 40 years. The comparison between the groups showed an obvious statistically significant difference (p = 0.000). The correlation with the other issues analyzed within the group of patients with hearing loss did not show statistical significance.

**Graph 7:**
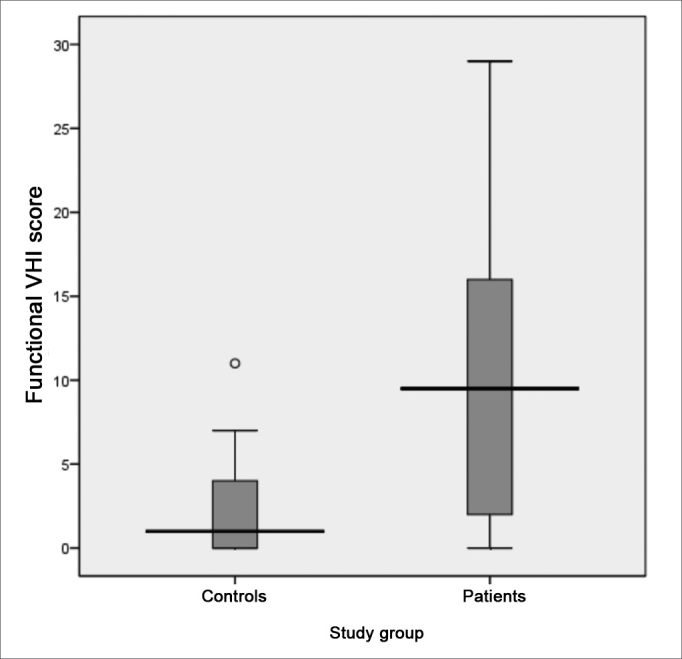
Distribution of the VHI functional subitem score between the groups studied (p = 0.000).

**Graph 8:**
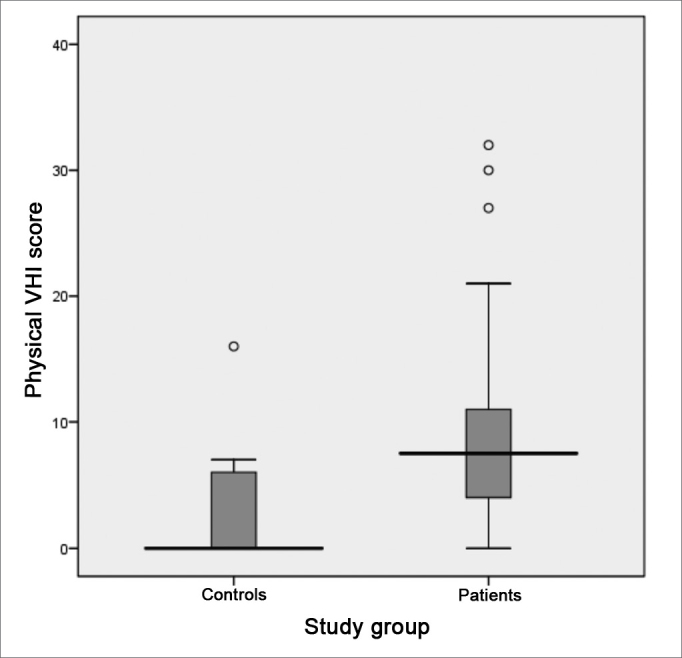
Distribution of the VHI physical subitem between the groups.

**Graph 9:**
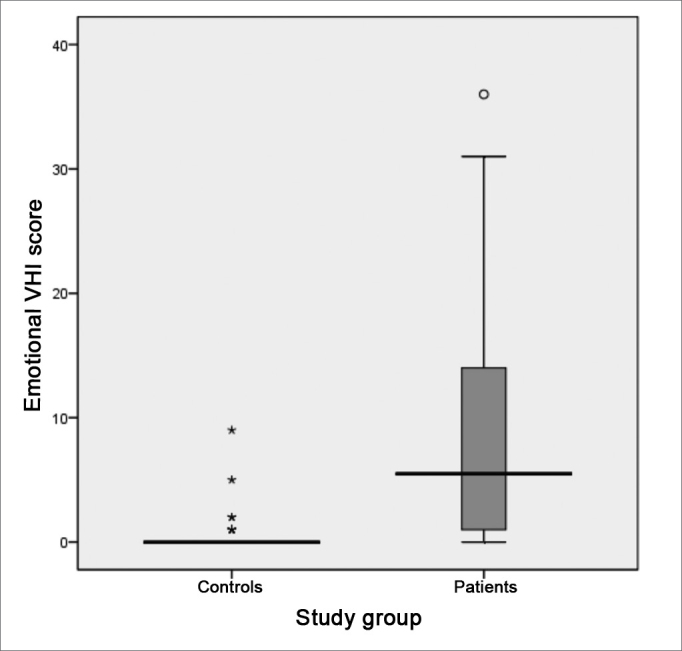
Distribution of the VHI emotional subitem score between the groups studied (p = 0.000).

**Graph 10:**
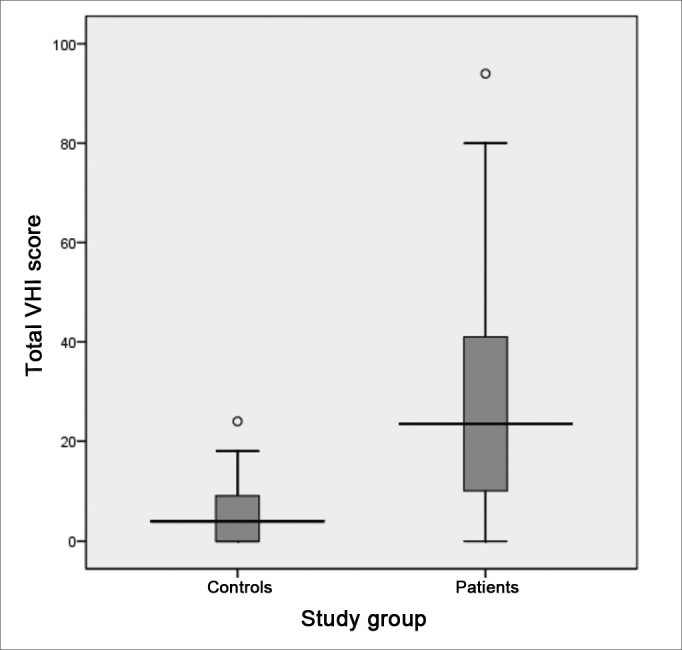
Distribution of the total VHI score between the groups studied (p = 0.000).

The VHI functional aspect showed a score median of 9.5 (SD = 8.5, variation between 0 and 29) and 1 (SD = 2.6, variation between 0 and 11) between patients and controls, respectively. The VHI physical aspect among patients showed a median score of 7.5 (SD = 8.1, variation between 0 and 32), while in the control group, the median was 0 (SD = 3.6, variation between 0 and 16). The emotional subitem had a median value of 5.5 (SD = 9.9, variation between 0 and 36) for the group of patients; and it was 0 (SD = 1.7, variation between 0 and 9) for the control group. The total score between the groups was 23.5 (SD = 23.1, variation between 0 and 94) and 4.0 (SD = 6.0, variation between 0 and 24) for patients and controls, respectively. The comparative analysis between the two groups showed a statistically significant difference for all VHI subitems, as well as for its total score: the functional, physical emotional and total aspects all showed a p-value of 0.000 (Graph 7, Graph 8, Graph 9, Graph 10).

The VHI emotional subitem showed a significant correlation with the tritonal mean values for both ears of the control group (left ear with r = +0.35, p = 0.032 and right ear with r = +0.35 and p = 0.031). There was also a strong correlation between the emotional subitem and the functional (r = + 0.723, p = 0.000) and physical (r = + 0.610, p = 0.000) subitems in the group of patients with hearing loss.

## DISCUSSION

Other studies were published along the years with the VHI in Portuguese. As previously alluded to, Jotz et al. showed the VHI accuracy in differentiating patients with and without dysphonia^14^. Tsuji et al. obtained significant results during the evaluation of patients under surgical treatment of adduction and spasmodic dysphonia^24^. Costa and Matias assessed the voice of elderly women without vocal complaints in order to study the impact of voice characteristic alterations in the elderly^16^. All the studies aforementioned, despite their small number, show the great utility of the VHI in its Portuguese version for the evaluation of voice-related disability and impairment.

We chose to include individuals between 18 and 60 years as a means to exclude the possibility of age-related vocal disorders - those associated with physiological vocal change and those associated with presbyphonia. The correlation between the total VHI score and the individual's age in the control group shows the trend towards a higher VHI score according to an increase in the age range found in the group of patients without hearing loss. Such data translates the natural trend of vocal pattern changes with age - expected in the control group since they are not affected by hearing loss.

The gender of the individuals included in this paper was not a factor influencing any of the other data analyzed. By the same token, a study published in 2006 showed VHI score data separately for the two genders^39^, also without statistically significant difference of VHI scores between the genders, strengthening the analysis hereby carried out, since the results obtained from the VHI would not have gender considered as a confounding factor on the questionnaire answers. The predominance of females in the samples analyzed is in agreement with data found in the literature^12,^^13,^^40^, enabling a comparative analysis between this study and the ones previously revised.

The tritonal averages were used in order to evaluate the group of patients with hearing loss for encompassing the 500; 1,000 and 2,000 Hz frequencies -concentrating most of the sound energy of complex sounds which characterize speech^41^. Such method was also used by Waldstein in an important paper published in 1990, where he discusses the effects of postlingual hearing loss on speech production^2^.

Based on Perkell's remarks on the role of hearing in vocal production1, in this paper we chose not to break the studied individuals down into ear sides. Not having any literature reference on the assessment of patients with unilateral hearing loss, nor comments on a possible laterality of the auditory feedback process, we included in this study only those patients with bilateral hearing loss.

The literature on the hearing loss influence on voice is restricted to the assessment of patients with profound hearing loss. By enrolling patients with moderate hearing loss and their analysis as a group we were able to show, in a statistically significant fashion, that it is possible to have vocal alterations and related complaints also in this group of individuals.

We also considered that, because of the major variability of responses obtained through using VHI in the group of patients (a variation of 94 points) and the subjective characteristic of the individual perception on vocal problems, there is no way to define a specific loss for patients with hearing loss, in other words, there is no way to describe a VHI score range such as a characteristic of patients with hearing loss, a comment which was also raised during the evaluation of laryngectomized patients with tracheoesophageal voice^34^. Later papers, grouping a larger and more similar group of patients with hearing loss, allowed the analysis of the vocal impairment according to the VHI score in each subgroup, increasing its efficacy.

The influence of the hearing loss duration is only speculated on vocal alterations^1^,^4^; nonetheless, in this study we did not find any significant correlation between the hearing loss duration time in the group of patients analyzed and the scores of the subitems and the VHI score. It is possible that the longer a person lives with some type of disability, the greater is the likelihood of this person adapting him or herself to that situation and live without greater losses of such status. We cannot exclude from this study, in which the duration of the hearing loss prior to the VHI evaluation varied between 1 and 40 years, the fact that those patients with longer hearing losses without medical help would live better with their vocal alterations. Although the statistical evaluation hereby done did not show any influence of the duration of hearing loss impacting the results obtained, later studies who were able to group a higher number of patients per period of hearing loss which could rule out this factor as a possible VHI scoring bias.

The results obtained by Jacobson in developing the VHI12 were groups according to the patient's perception on the intensity of their vocal problem in categories defined as normal voice; mild, moderate and severe dysphonia. The total average VHI scores obtained in each one of these categories were 33.69 for normal and mild (grouped for analysis), 44.37 for moderate and 61.39 for severe. In the present study, the total score of the patients with hearing loss showed a median value of 23.5, reasonably comparable with the group of mild dysphonia or with normal voice in the study by Jacobson; comparing the functional, physical and emotional subitem, his scores in this study are comparable only to the group with normal voice and mild dysphonia. The group studied by Jacobson in the development of the VHI was made up of patients with diagnosed laryngeal diseases, while the patients here analyzed had, necessarily, videolaryngoscopy without alterations. Such fact can justify the total VHI scores being less in our paper when compared to the group classified as mild dysphonia or no dysphonia by Jacobson.

In the study published by Guimarães and Abberton we applied the same previous comments when comparing the results with those from Jacobson in 1997. The presence or absence of dysphonia was considered as defining the groups analyzed (average VHI total score of 34.4 and 10.5, respectively)^13^, near the ones shown here. When we compare to the paper by Jotz et al.^14^, we see results very similar to the ones here presented (median values of 20.5 for the dysphonia group and 4.0 for the group without dysphonia), which corroborates the presence of voice-related problems in patients with hearing loss starting at a moderate level, such as the ones evaluated here.

The total VHI score median value for the control individuals was 4.0 (SD: 6.0), matching the results from papers who assessed control groups made up of individuals without vocal alterations, even when considering the different distribution values used in the papers we analyzed. Very few studies involved the use of a control group when assessing the VHI in specific clinical situations: stressing the findings by Peeters in 2004, showing a total score of 2.331 for controls. The controls used by Khaintan had total mean score of 6.219, similar to findings reported by Pribuisiene (4.1 and 4.66)20. Guimarães and Abberton^13^ showed in a normal voice group a mean value of 10.5 in the total score, while Jotz et al.^14^ reported very similar results to the ones hereby presented, with a group without dysphonia reporting average total score of 4.0.

As to the results regarding the scores obtained from the group of patients with hearing loss, similar results to total VHI were also published by Behrman in 200429, in assessing patients with benign laryngeal alterations such as polyps, intracordal cysts and nodules. The median found in that study for the total VHI score was 30, not so far from the one found in the present paper (23.5). Similar scores were also found by Loughran in 2005 in patients submitted to endoscopic surgery or radiotherapy for initial laryngeal cancer (22.2 and 25.4, respectively)^32^ by Jepsen in 2003^42^ in patients under postoperative of supraglottic laryngeal cancer (27.7) and in patients with laryngeal cancer submitted to radiotherapy (29) in 2003 by Meleca^33^. Other comparable groups found in the literature were of patients with gastroesophageal reflux disorder^19^ (mean total score of 21) and patients with laryngopharyngeal reflux (29.4 and 23.1 for women and men, respectively)^21^. The total scores of patients with laryngopharyngeal reflux were 28.14 in a study led by Pribuisiene et al. in 2006^20^.

The results obtained in patients with presbyphonia and post-radiotherapy of the T1a glottic cancer without disease recurrence^43^ are similar to the ones found here for the group with hearing loss. Total mean scores found in the study mentioned were 29.9 (presbyphonia) and 28.5 (post-radiotherapy), very close to the total VHI score median value found here (23.5). It is possible to compare such groups and patients with hearing loss in relation to the subjective evaluation of their voice use.

The papers on patients with spasmodic dysphonia (in adduction and abduction) show results with total VHI scores varying between 63.46 and 102^16,^^22,^^23^, more than twice the median found in the present study, a trend we can clearly see in the scores of each subitem^23^. Patients with unilateral vocal fold paralysis also showed higher scores than those in the hearing loss group, varying between 87.9 and 62.8. We have also to consider that such patients also had other underlying diseases, such as the case of patients with head and neck cancer or those at terminal state of the disease^44^. It is possible to have the influence of the general health status on the perception of associated vocal problems, both for overrating as well as for the negligence of the vocal symptoms. In the present study, the auditory alterations did not cause fast or obvious vocal changes in a short period of time, and this somehow may reduce its influence on the final score.

As we compare the results obtained in the VHI subitems and total scores to the studies published on the use of the same questionnaire in other clinical situations, we can see that the scores of the patients with hearing loss are usually lower than the ones already published in the literature. Thus, the disability caused by the hearing loss could be included as the ones with the lowest impact when compared to the other ones analyzed in the aforementioned studies.

The analysis of the total and partial scores between the control and hearing loss groups showed a statistically significant difference (p = 0.000), which says that the patients with hearing loss had significant levels of voice-related disabilities when compared to the groups, corroborating the presence of vocal changes in these patients. The control group in this paper was strictly built, reinforcing even further the VHI score differences between the groups. The bivariate analysis of the VHI subgroup scores showed a positive correlation between each one of the subitems, saying that in the group of hearing loss patients there are not major differences as to the vocal disability pattern, in functional, physical or emotional aspects, which could be shown in the present study.

As previously stated, objective methods of voice analysis, with vocal spectrograph, bear important information on the severity of the vocal involvement when compared to normal voices; however, they fail in indicating why and how patients with similar alterations have different social and personal impairment. The VHI questionnaire represents an important development on this issue. The comparison between such methods and VHI may not show strong correlations; however, it does not cancel out the analysis done in this paper because here we considered the level of perception of the vocal disability, which is a subjective parameter. The paper from Hsiung also shows and corroborates this fact, because they did not show a correlation between the objective method (vocal spectrograph) and the subjective one (VHI)^45^.

In this paper, we obtained a strong correlation between the VHI subitems from the hearing loss patients, indicating that the voice alteration affects multiple aspects of these individuals, including functional, physical and emotional aspects, besides the economic, affective and others. Thus, we must consider that the symptoms from a dysphonic syndrome include not only hoarseness or voice asthenia, for example, but also other issues which deeply influence the lives of patients and which are lived by each person in a different way.

The patients hereby analyzed were collected among those referred to a tertiary care Otolaryngology service, causing possibilities of sampling bias because the population seen at our place is already selected from a population with hearing loss. Considering this selection bias, we believe that such share of the patients who came to a university care center would be a group already convinced of the need to treat their hearing problem. There is not a way to rule out a greater concern these patients have in relation to their hearing loss, including the vocal disorders, as already mentioned by other authors^22^. Therefore, there is a possibility of greater bias associated to the VHI questionnaire these patients answer. Such problem may be corrected in future studies by including a greater number of patients among those not initially referred to wear a hearing aid.

## CONCLUSION

Through the results obtained in this study, we can state that patients with bilateral hearing loss starting from a moderate level have higher scores than patients with their hearing within normal ranges in a statistically significant fashion. Based on this analysis, the researcher points to this trend towards a greater social or economical disadvantage stemming from the disability or physical impairment specifically associated with the vocal alterations caused by such hearing loss.
